# Online Multi-Domain Geriatric Health Screening in Urban Community Dwelling Older Malaysians: A Pilot Study

**DOI:** 10.3389/fpubh.2020.612154

**Published:** 2021-01-14

**Authors:** Deepa Alex, Adhhani Binti Fauzi, Devi Mohan

**Affiliations:** ^1^Jeffrey Cheah School of Medicine and Health Sciences, Monash University Malaysia, Bandar Sunway, Malaysia; ^2^Global Public Health, Jeffrey Cheah School of Medicine and Health Sciences, Monash University Malaysia, Bandar Sunway, Malaysia

**Keywords:** older population, Asia, online, frail, geriatric syndromes

## Abstract

**Introduction:** With a rapidly aging population, the Malaysian health care system needs to develop solutions to address the lack of resources that are required for the assessment of the older person. The complex nature of geriatric syndromes coupled with the occurrence of multiple comorbid illnesses with aging, make geriatric assessment a resource intensive process. Digital health solutions could play an important role in supporting existing health care systems, especially in low and middle income countries, with limited speciality services in geriatrics.

**Objective:** This is a pilot study aimed at screening for geriatric syndromes through self-administered online surveys in urban community dwelling older Malaysians and assessing the pattern of geriatric syndromes in relation to the frailty status of the study participants.

**Methods:** This is a cross-sectional pilot study conducted between July-September 2020. Community dwelling adults aged 60 years and over were invited to take part in an online survey. Information on sociodemographic variables, comorbidities, and the self-reported results of geriatric syndromes (frailty, sarcopenia, anorexia of aging, urinary incontinence, falls, and cognitive impairment), were collected through the survey.

**Results:** Data was collected for 162 participants over a period of 2 months. The mean (SD) age of the respondents was 66.42 (5.25) years with 64.9% females. Majority of the respondents were of Chinese ethnic origin (67.9%) and had tertiary level of education (75.9%). The average time taken by participants to complete the survey was 16.86 min. Urinary incontinence was the highest reported geriatric syndrome (55.1%) followed by falls (37.6%), anorexia of aging (32.8%), cognitive impairment (27.8%), and sarcopenia (8.3%). Frailty was detected in 4.5% of the study population. Loss of weight in the previous year was the highest reported component of the frailty assessment tool. The presence of sarcopenia, anorexia of aging, poor/fair self-rated health, urinary incontinence, and multimorbidity were significantly higher in older adults who were frail or prefrail.

**Conclusion:** Screening for geriatric syndromes through online surveys is a feasible approach to identify older adults in the community who are likely to benefit from geriatric assessment. However, the demographic profile of the older population that are accessible through such digital platforms is limited.

## Introduction

Geriatric syndromes are multifactorial conditions that are common in older adults. They are distinct from organ specific disease conditions due to their complex pathogenesis, presence of multiple risk factors and atypical clinical presentation (1). Geriatric syndromes include but are not limited to dementia, delirium, falls, incontinence, pressure ulcers, malnutrition, frailty, and sarcopenia. Frailty is one of the newer geriatric giants and is characterized by decreased ability of the body's physiologic response to maintain homeostasis during periods of stress (2). Frailty is not only associated with poor health outcomes such as increased dependency, hospitalizations, and mortality but is also a major cause of economic burden (3, 4). Frailty along with other geriatric syndromes are best identified through a Comprehensive Geriatric Assessment (CGA). Comprehensive geriatric assessment (CGA) is a multidimensional process which identifies the medical, social, and functional needs of older adults, for the development of a coordinated care plan to meet those needs (5). CGA conducted in a clinical setting takes 1–2 h on an average (6). It is usually undertaken by an interdisciplinary team comprising clinicians, nurses, occupational, and physical therapists. The Rapid Geriatric Assessment (RGA) is a tool developed in order to conduct a quick screening of four geriatric syndromes which include frailty, sarcopenia, anorexia of aging, and cognitive impairment (7).

In Malaysia, the aging population is rapidly increasing while trained geriatricians are still limited in number (8). Geriatric training was recently included as a core component of the undergraduate medical curriculum (9). Therefore, primary care clinicians have limited knowledge on the detection and management of geriatric problems. Older adults who do access geriatric units are by way of referral and are mostly those who require acute care with complex care needs. The role of early detection and prevention of the geriatric giants such as frailty and dementia are heavily emphasized in developed countries where older adults comprise a significant proportion of the overall population (10). As of 2020, 7% of the Malaysian population are aged 65 years (11) and above, and this is expected to double by 2035 (12).

A study in older urban community dwelling Malaysians found that 67.7% of the study population were pre-frail and 5.7% were frail (13). As a frailty is a potentially reversible condition if identified early, the high prevalence of pre-frailty in the Malaysian population indicates an urgent need to address the factors contributing to its development at an early stage. The occurrence of geriatric syndromes can have a major impact on the quality of life for older adults (14). However, in the current health care system, majority of these conditions are identified and managed during hospitalization of older adults, by which time most of them are already dependent for care. Therefore, it is crucial to set up systems and solutions that support screening and early detection of geriatric syndromes at a community level (15). This will enable the identification of those individuals who can benefit from targeted geriatric assessment and management.

Current healthcare approaches are primarily disease centered rather than person centered (16). Time per patient is a critical component as current healthcare settings are geared toward seeing more patients in a limited period of time. This approach is not well-suited for the aging population due to the presence of multiple comorbidities, increasing complexity, and atypical presentation of disease. Gathering information from an older patient or caregiver requires a substantial amount of time due to the reasons mentioned and takes up a significant portion of the clinical visit. One potential solution to this problem is to allow patients to provide their information prior to the actual visit, that can be utilized by the clinician as a valuable source of information for clinical decision making and management. Data provided by patients through an online platform, which is accessible to the healthcare provider, will also assist the clinician to focus on areas of priority for the older individual while using consult time more efficiently.

There is limited data on the awareness and use of digital health tools among the older Malaysian population. Studies suggest that social media is widely used by older Malaysians, primarily for the purpose of communication with friends and family, and majority of them use smartphones to access social media (17). The use of online platforms for the purpose of health screening and assessments in older adults have been studied in developed nations, but there is limited evidence from Asian countries (18). Therefore, a pilot study was conducted to screen for geriatric syndromes through self-administered online surveys in urban community dwelling older Malaysians and to assess the patterns of geriatric syndromes in relation to the frailty status of the study participants.

## Methods

This was a pilot cross-sectional study conducted through an online survey from July–September 2020. Participants for the study were recruited by convenience sampling. The inclusion criteria for the study were Malaysians citizens aged 60 years and above who were willing to participate in the online survey. Individuals aged 59 years and below were excluded from the study. Recruitment posters were circulated in closed social media community groups for older Malaysians in the Klang valley, which is an urban conglomeration in Malaysia that includes Kuala Lumpur and the adjoining state of Selangor. Information about the study was disseminated to seven organizations for older adults including non-profit organizations and healthcare organizations for senior citizens. Respondents were directed to an online link with the explanatory statement and a consent form. Those who consented were then directed to the online survey. The survey questionnaire was generated using Qualtrics software, Version [September, 2020] Copyright © [2020] (19). The study was approved by the Monash University Human Research Ethics Committee 2020-21334-45510.

The survey collected data on sociodemographic factors, comorbid illnesses, medications and geriatric syndromes. Information on geriatric syndromes such as frailty, sarcopenia, nutrition and cognition, falls, urinary incontinence, and sensory impairments was collected through the online questionnaire.

### Frailty

Frailty was assessed using the FRAIL scale. It consists of 5 items: fatigue, unable to climb 1 flight of stairs, unable to walk 1 block, presence of 5 or more chronic illnesses and loss of weight of more than 5% in the past 1 year (20). Loss of weight was calculated as the difference between self-reported weight 1 year ago and current self-reported weight. A score of 3 or above over 5 indicates the presence of frailty. A score of 1–2 over 5 indicates pre-frailty and absence of any of the items indicates a robust state.

### Sarcopenia

Sarcopenia was assessed using the SARC-F tool and consists of 5 components: strength, assistance in walking, rise from a chair, climbing stairs, and falls in the past 1 year (21). The scores range from 0 to 10 and a score of 4 or higher indicates sarcopenia.

### Nutritional Status

The Simplified Nutritional Appetite Questionnaire (SNAQ) was used to assess appetite. It has 4 items: appetite, food taste, feeling of satiety, and number of meals consumed in a day (22). Possible scores range from 4 to 20. A score of ≤ 14 over 20 is indicative of anorexia and is predictive of at least 5% weight loss in the following 6 months.

### Cognition

Cognitive impairment was assessed using the Alzheimer's Dementia Screening Interview (AD8). The AD8 comprises of 8 questions that test for memory, orientation, judgment, and function and can be self-administered. The participant rated AD8 has been validated for use in an Asian population (23).

### Falls

The presence of a fall in the preceding 1 year was ascertained by the question, “Have you fallen in the past 12 months?” The number of falls over the past 1 year is a component of the SARC-F tool. A fall was defined as unintentionally coming to rest on the ground, floor, or lower level (24). Further data on falls requiring hospitalization, and fear of falling was captured in the questionnaire.

### Urinary Incontinence

The presence of urinary incontinence was identified by inquiring about symptoms of stress and urge incontinence through the questions “Do you ever wet yourself when you cough or strain?” and “Do you ever wet yourself before you reach the toilet?” respectively. The presence of symptoms of either stress or urge incontinence or both were considered as the presence of urinary incontinence.

### Multimorbidity

The number of comorbid illnesses was summed and calculated from a total of 13 self-reported medical conditions including hypertension, diabetes mellitus, dyslipidemia, heart disease, arrhythmias, arthritis, cancer, Parkinson's disease, stroke, chronic lung disease, cirrhosis, osteoporosis, and thyroid disease. Multimorbidity was defined as the presence of 5 or more chronic illnesses.

### Self-Rated Health

Response options for self-rated health ranged from “excellent” to “poor.” Self-rated health was then classified into 2 categories, those with poor/fair self-rated health and those with good to excellent self-rated health.

### Sensory Impairment

Vision and hearing impairments were assessed by self-reported impairments in vision and hearing, respectively.

Data analysis was carried out using SPSS software (v.24) (25). Continuous variables were presented as mean (standard deviation) while categorical variables were presented as frequencies (percentages). All variables were calculated as categorical data except for age which was taken as a continuous variable. Bivariate analysis was performed using the Chi-square test for categorical variables and the Mann-Whitney *U*-test for continuous variables with non-normal distribution. A *p* < 0.05 was taken as statistically significant.

## Results

A total of 162 participants answered the online survey. Fifteen duplicate responses, two potential bot responses (detected by the Qualtrics software program), seven incomplete responses and four participants who were non-eligible on the basis of age, were excluded from final data analysis. Complete data was available on 134 participants. Participants took an average time of 16.86 min to complete the survey. The mean (SD) age of the respondents was 66.42 (5.25) years. The demographic characteristics of the study participants can be found in Table 1. The major proportion of the study participants belonged to the age group 60–70 years (82.9%). Majority of the respondents were female, had tertiary level of education and were of Chinese ethnic origin. Twenty respondents (15%) were living alone and 103 respondents (77%) were retired or unemployed.

**Table 1:** Baseline characteristics of participants.

**No**	**Variable**	**Frequency, (percentage)**
1	**Age groups**	
	60–65	66 (49.3)
	65–70	45 (33.6)
	70–75	13 (9.7)
	>=75	10 (7.5)
2	**Gender**	
	Male	47 (35.1)
	Female	87 (64.9)
3	**Ethnicity**	
	Chinese	91 (67.9)
	Malay	23 (17.2)
	Indian	17 (12.7)
	Others	3 (2.2)
3	**Education**	
	Primary	7 (5.2)
	Secondary	32 (23.9)
	Tertiary	95 (75.9)
4	**Employment status**	
	Working, part time or full time	31 (23.1)
	Retired/Unemployed	103 (76.9)
5	**Marital status**	
	Married	93 (69.4)
	Divorced/Single/Widowed	41 (30.6)
6	**Living alone**	
	No	114 (85.1)
	Yes	20 (14.9)
7	**Smoker/Ex-smokers**	19 (14.2)
8	**Alcohol use**	29 (21.6)

**Figure 1:**
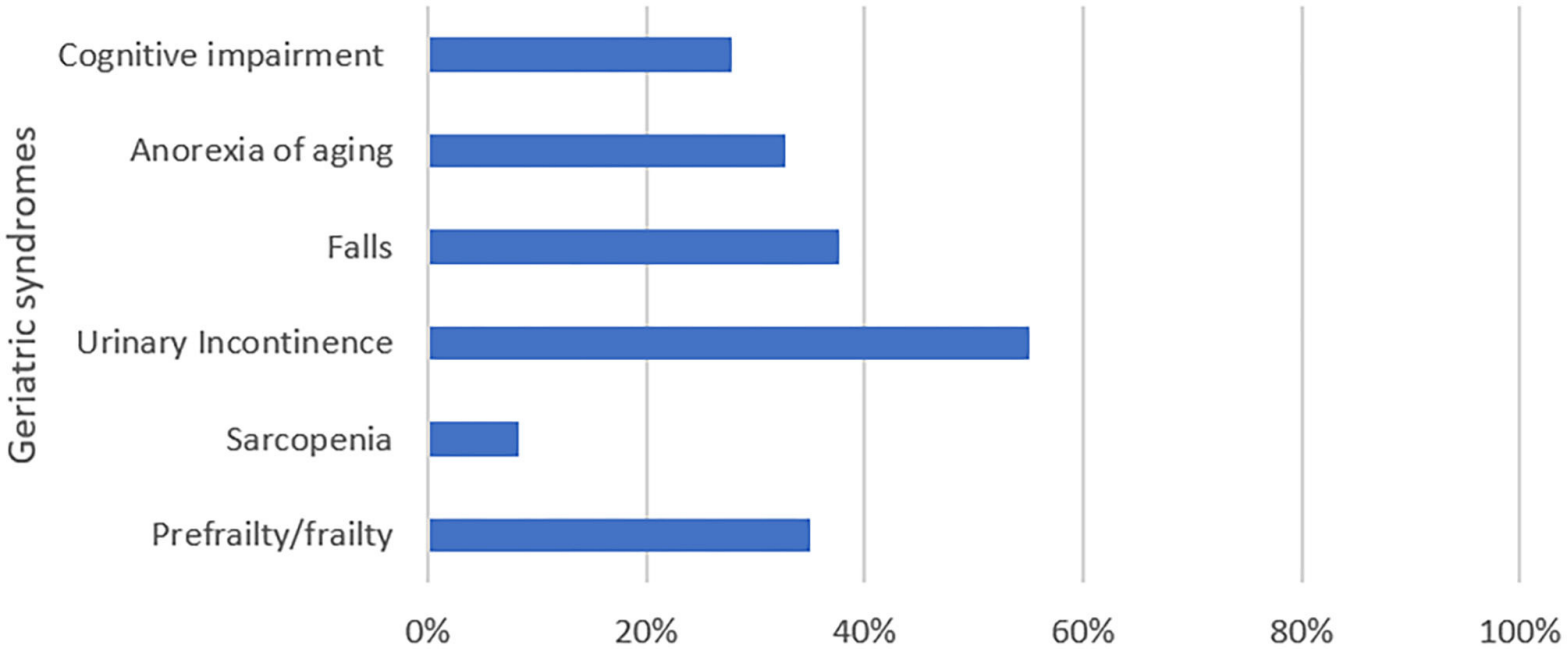
Geriatric syndromes reported in study population.

Figure 1 shows the distribution of geriatric syndromes reported in the study population. Frailty was detected in six participants (4.5%), 41 participants (30.6%) were pre-frail and 87 respondents (64.9%) were robust older adults. Sarcopenia was detected in 11 respondents (8.3%). Anorexia of aging was found in 44 participants (32.8%) while cognitive impairment was reported by 37 participants (27.8%). Fifty participants (37.6%) had experienced at least 1 fall in the past year. Fear of falls was reported by 101 (79.5%) participants. Out of 118 respondents (88.1%) who responded to questions on urinary incontinence, 65 (55.1%) were found to have symptoms of stress or urge incontinence or both. Vision impairment was reported by 63 participants (48.8%) while hearing impairment was reported by 27 respondents (22%). Table 2 shows the sociodemographic distribution of geriatric syndromes according to age and gender.

**Table 2:** Geriatric syndromes according to age and gender distribution.

		Age categories^*^					Gender categories^*^		
		**60–65 years**	**65–70 years**	**70–75 years**	**≥75 years**	***p-*value**	**Male**	**Female**	***p-*value**
1	Pre-frailty/Frailty	23 (34.8)	13 (28.9)	6 (46.2)	5 (50)	0.487	16 (34)	31 (35.6)	1.00
2	Sarcopenia	4 (6.2)	4 (8.9)	1 (7.7)	2 (20)	0.527	1 (2.1)	10 (11.6)	0.096
3	Urinary Incontinence	32 (53.3)	24 (58.5)	5 (41.7)	4 (80)	0.494	19 (50)	46 (57.5)	0.553
4	Falls	26 (40)	18 (40)	4 (30.8)	2 (20)	0.604	17 (36.2)	33 (38.4)	0.853
5	Anorexia of aging	20 (30.3)	16 (35.6)	3 (23.1)	5 (50)	0.524	16 (34)	28 (32.2)	0.849
6	Cognitive impairment	16 (24.6)	14 (31.1)	2 (15.4)	5 (50)	0.259	14 (29.8)	23 (26.7)	0.840

* *Frequency (Percentage)/p-value derived by chi square*.

The presence of frailty, sarcopenia, incontinence, anorexia of aging, and cognitive impairment is higher among older adults aged 75 years and above, in comparison to other age groups. The pattern of the geriatric syndromes was analyzed across various strata of frailty. Table 3 shows this comparison.

**Table 3:** Comparison between categories of frailty.

**No**	**Variable**	**Robust**	**Pre-frail/frail**	***p*-value**
1	Age (median, IQR)	66, 6	66, 7	0.486^a^
2	Female gender	56 (64.4)	31 (66)	1.00^b^
3	Self-rated health (Poor/fair)	15 (17.2)	21 (45.7)	0.001^b^
4	Multimorbidity	0 (0)	4 (8.5)	0.014^b^
5	Vision impairment	40 (47.6)	23 (51.1)	0.716^b^
6	Hearing impairment	17 (22.1)	10 (21.7)	1.00^b^
7	Sarcopenia	3 (27.3)	8 (72.7)	0.016^b^
8	Reported falls in past 1 year	28 (32.6)	22(46.8)	0.134^b^
9	Presence of fear of falls	61 (76.3)	40 (85.1)	0.263^b^
10	Presence of incontinence	36 (46.2)	29 (72.5)	0.011^b^
11	Presence of cognitive impairment	21 (24.4)	16 (34)	0.311^b^
12	Anorexia of aging	21 (24.1)	23 (48.9)	0.007^b^

a *Mann Whitney-U-test for continuous variables*.

b *Chi-square test for categorical variables*.

**Figure 2:**
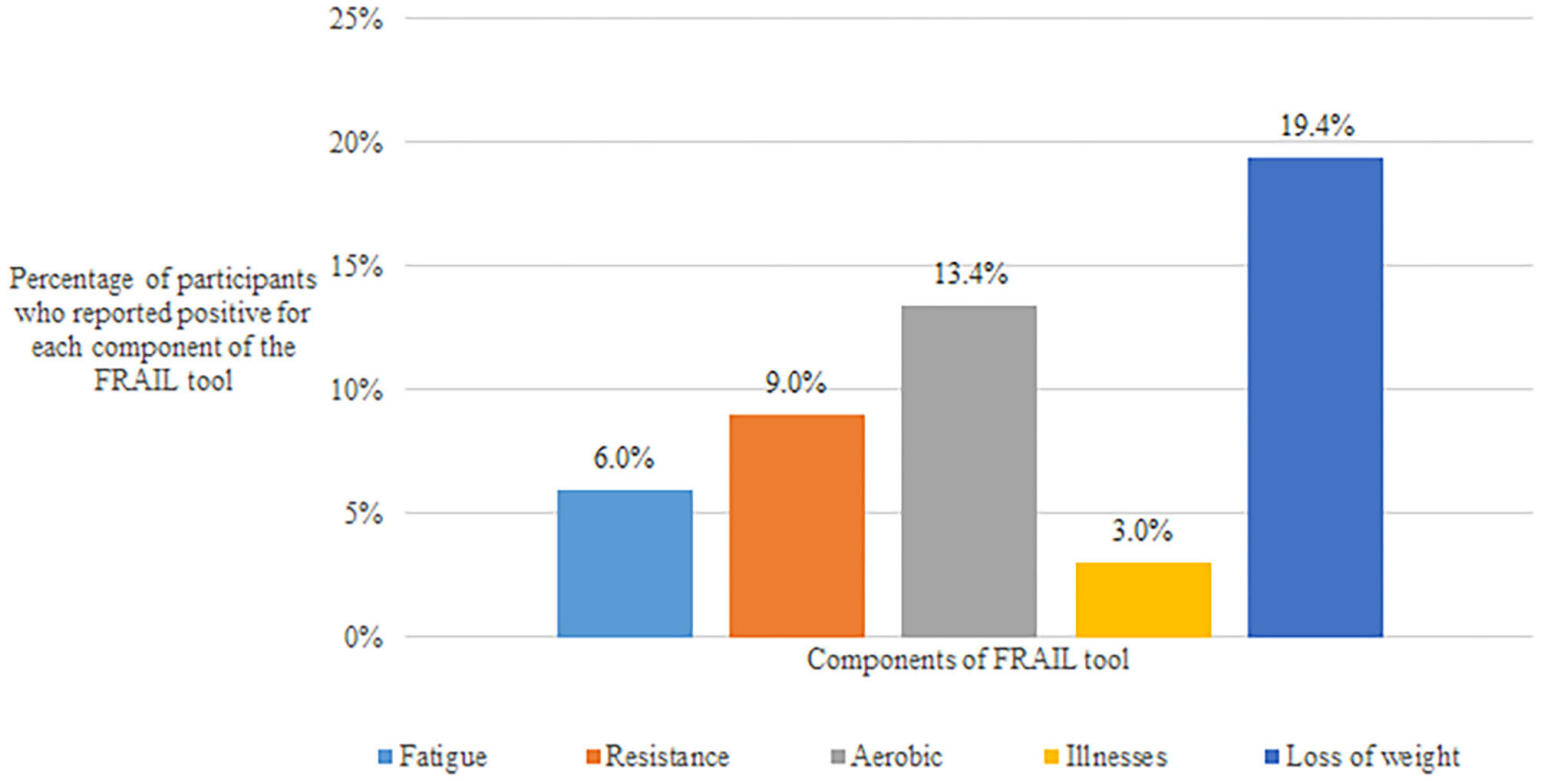
Distribution of components of FRAIL tool in the study population.

The presence of sarcopenia, anorexia of aging, poor/fair self-rated health, urinary incontinence, and multimorbidity were significantly higher in older adults who were frail or prefrail. The FRAIL scale was used for the detection of frailty in this study. Figure 2 shows the distribution of the individual components of FRAIL tool. Loss of weight of >5% was the highest reported component of the FRAIL scale.

## Discussion

Conducting a pilot study on screening for geriatric syndromes through an online survey in Malaysia has brought out several important findings. The study was conducted over a period of 2 months, over which we received 162 respondents in the first 40 days of the study. Complete data on outcomes of interest was available in 90% of the responses. The average duration to complete the online survey was 16.86 min. This indicates that it is feasible to carry out data collection through an online platform over a stipulated period of time. Respondents have undertaken self-reported online surveys for the purpose of geriatric screening, from the comfort of their own environments, which could provide valuable information for the clinician.

Majority of the study participants were those with tertiary level of education and predominantly were of ethnic Chinese origin. This may be indicative of the segment of the population that actively access community groups through social media and online platforms. This is also a limiting factor of our study, as there is no external validity and therefore, our findings cannot be extrapolated to the general population. The organizations that were approached for this study were mainly community groups for older adults with an online presence. Some of the groups had regular activities and forums conducted online and face to face. Other online platforms were primarily used for information dissemination meant for older Malaysians. Digital tools and interventions developed for older adults in Malaysia need to be designed considering the demographic profile of the target population. At the same time, future interventions to improve use and access to such digital tools need to include those older adults who have limited exposure to such platforms.

Frailty is a state of increased vulnerability to stressors due to an accumulation of deficits across multiple physiologic systems (26). Frail older adults are at an increased risk of negative health outcomes such as falls, physical limitation, hospitalization, and mortality (27). There are multiple instruments to detect frailty such as the Physical Frailty phenotype and the accumulation deficit index etc. (28). Yet, this condition is often overlooked by clinicians and is seldom addressed at an early stage. This could be because of its complex nature of presentation and also due to lack of awareness in the treating physician. Frailty is a continuum that is categorized into 3 stages: robust, pre-frail and frail. The FRAIL scale is a simple screening questionnaire to detect frailty which has the added advantage of a management algorithm for each of its individual components (29). Using the FRAIL tool, 4.5% of our study population screened positive for frailty and 30.6% were found to be pre-frail. Among the individual components of the FRAIL scale, loss of weight in the past 1 year was the highest reported by our study participants. Interestingly, about one third of our study population were found to have poor appetite and at risk for future loss of weight through the Simplified Nutritional Appetite Questionnaire. Anorexia of aging is common in the geriatric population but it is often attributed to the normal aging process (30). It is a risk factor for the development of frailty. This indicates that anorexia of aging is a significant problem in older Malaysians and it requires regular screening and appropriate interventions. However, our study design was cross-sectional and therefore follow up data was not available for the participants. This is an interesting finding that can contribute to the development of appropriate interventions for frailty prevention for older adults in Malaysia. While physical exercise has been proven to play a pivotal role in frailty management, nutritional interventions are currently an area of active research (31). Further research is required to understand the nutritional component of frailty and how it can play a role in frailty prevention and management.

Sarcopenia is defined as the progressive loss of muscle mass and function (32). It is one of the newer geriatric syndromes and is associated with adverse health outcomes such as falls, fractures, hospitalizations and increased mortality (33). The SARC-F is considered to be a suitable tool for screening for sarcopenia in the community (21). Sarcopenia was found to be significantly higher among the pre-frail/frail study participants. This is similar to findings from other studies as sarcopenia is considered to a key component of physical frailty. Participants of Malay ethnicity and females were found to have higher rates of sarcopenia when compared to their counterparts but these findings were not significant. This could be due to the low power of the study due to small sample size. Falls is one of the components of the SARC-F. 37.3% of the study participants reported that they had at least 1 fall over the past 1 year. Fear of falling was detected in 79.5% of the respondents. Women were found to have significantly higher levels of fear of falling when compared to men. There was no significant difference between fallers and non-fallers with respect to the fear of falling.

Cognitive impairment was assessed using the Alzheimer's Dementia Screening Inventory (AD8) that was self-administered. 27.8% respondents were found to have cognitive impairment. The participant rated AD8 is useful to gain a preliminary understanding of an individual's cognitive status (23). Considering the fact that our study participants are able to access and answer questions through an online survey, the findings suggest that these might be individuals with early cognitive changes but are functionally independent. Those detected to have cognitive impairment might be candidates for formal cognitive assessment and close follow up in the future. On the other hand, it would be highly unlikely for individuals with severe cognitive impairment to access such online surveys. Therefore, the role of screening through online platforms is better applicable for the early detection of cognitive impairment in older individuals.

Urinary incontinence is a frequently under-reported and undermanaged problem in the geriatric population. This could be due to social embarrassment or cultural barriers to discuss such issues with the healthcare provider. Out of those who agreed to answer questions on incontinence, 55% of the study population reported the presence of stress or urge incontinence which is much higher than the prevalence of urinary incontinence of 30.8% reported in an urban population in Malaysia (34). Answering sensitive questions through a survey may have prompted patients to be more open about their symptoms. Frail and prefrail older adults were more likely to report urinary incontinence when compared to robust older adults. Timely monitoring and effective management of this condition could have a role in delaying frailty and its consequences in older Malaysians.

Self-rated health is a simple measure of the overall subjective health status of an individual. It has been found to be useful in identifying vulnerable groups of older people who can benefit from targeted interventions (35). Self-rated health has also been shown to be useful in predicting institutionalization in community dwelling elderly in longitudinal studies (36). Our study findings show that self-rated health reported as “poor” or “fair” is significantly higher in pre-frail/frail individuals when compared to individuals in the robust category. Self-rated health is a simple measurable tool which has been demonstrated to be a valid predictor of chronic morbidity and mortality (37). Such findings are useful to inform resource planning and care delivery services in health care systems of developing countries such as Malaysia.

Our study has a few limitations. As data was self-reported and collected through an online tool, there could be errors in data reporting that could affect the accuracy of data. However, every effort was made to minimize false data reporting by enabling multiple error proofing options that are available through the Qualtrics software. Furthermore, responses that were flagged as duplicate responses or bot responses were excluded from the final data analysis. Information on follow up outcomes were not available due to the cross-sectional design of the study. As the screening was based on self-reported data through an online platform, it will have to validated against clinician delivered geriatric assessments. Moreover, the tools included in the questionnaire require validation for use through an online survey. The current study may not have been successful in reaching out to a significant proportion of frail older adults who are dependent and have complex care needs due to limited access or inability to use digital platforms. The respondents of the online survey in our study are likely to be older adults who have the physical and cognitive abilities to use digital gadgets such as computers and handphones which indicates a reasonably good state of overall health. As depression was not assessed, this could have led to a possible bias in the other assessments reported in the study. Yet, geriatric syndromes reported in our study population were high which suggests that the actual magnitude of the problem could be much larger in the general population. Lessons learnt from this study can be used to inform the development of future digital health interventions for the older population.

The development of digital tools for health care are all the more relevant in the wake of the Covid-19 pandemic. Older citizens are increasingly concerned about visiting medical facilities for the management and follow up of their pre-existing ailments. Due to national lockdowns, there have been physical restrictions to travel to health facilities. Moreover, as the aging population are highly vulnerable to infections in the current situation, there is a conscious or unconscious delay in getting to hospitals, especially for detection and diagnosis of new medical conditions. This can be detrimental to the overall health of older people. Frail older adults are the most affected in times of global crisis such as the Covid-19 pandemic (38) and recognizing geriatric syndromes early has never been more relevant. The study has highlighted that there is an urgent need for researchers and policy makers to identify effective methods for early detection of geriatric syndromes in older Malaysians.

## Conclusion

This study has shown that screening for geriatric syndromes through self-administered online surveys is feasible to a certain extent, in urban Malaysian settings. Clinically valuable information can be collected using such tools that can be utilized by healthcare providers for better diagnosis and management. Future studies will be required to validate the use of online surveys for geriatric screening against clinical assessments. This method of screening older adults online can substantially reduce the burden on the health care system by more efficient use of existing resources. The study showed that the main challenge in using online tools for healthcare screening is to ensure accessibility and effective use by all segments of the older population.

## Data Availability Statement

The datasets presented in this article are not readily available because due to concerns about loss of fidelity of personally identifiable data, the dataset is currently not available publicly. However, parts of the dataset will be released anonymised through written requests submitted to the corresponding author. Requests to access the datasets should be directed to Deepa Alex, deepa.alex@monash.edu.

## Ethics Statement

The studies involving human participants were reviewed and approved by Monash University Human Research Ethics Committee (2020-21334-45510). The patients/participants provided their written informed consent to participate in this study.

## Author Contributions

DA and DM designed the study and contributed to writing of the manuscript. DA and AF were responsible for the conduct of the study and carried out the data analysis. All authors reviewed and approved the final submitted version.

## Conflict of Interest

The authors declare that the research was conducted in the absence of any commercial or financial relationships that could be construed as a potential conflict of interest.
